# Supplementary material for: A Meta-Analysis on Quantitative Calcium, Phosphorus and Magnesium Metabolism in Horses and Ponies
Source: Animals (Basel). 2024 Sep 25;14(19):2765. doi: 10.3390/ani14192765 (PMC11475699; doi:10.3390/ani14192765)
Supplement: Supplementary file 1 [file animals-14-02765-s001.zip › animals-3171872-supplementary.pdf]

Figure S1. Flow diagram for study search and selection based on Preferred Reporting Items for Systematic Reviews and Meta-Analyses (PRISMA).

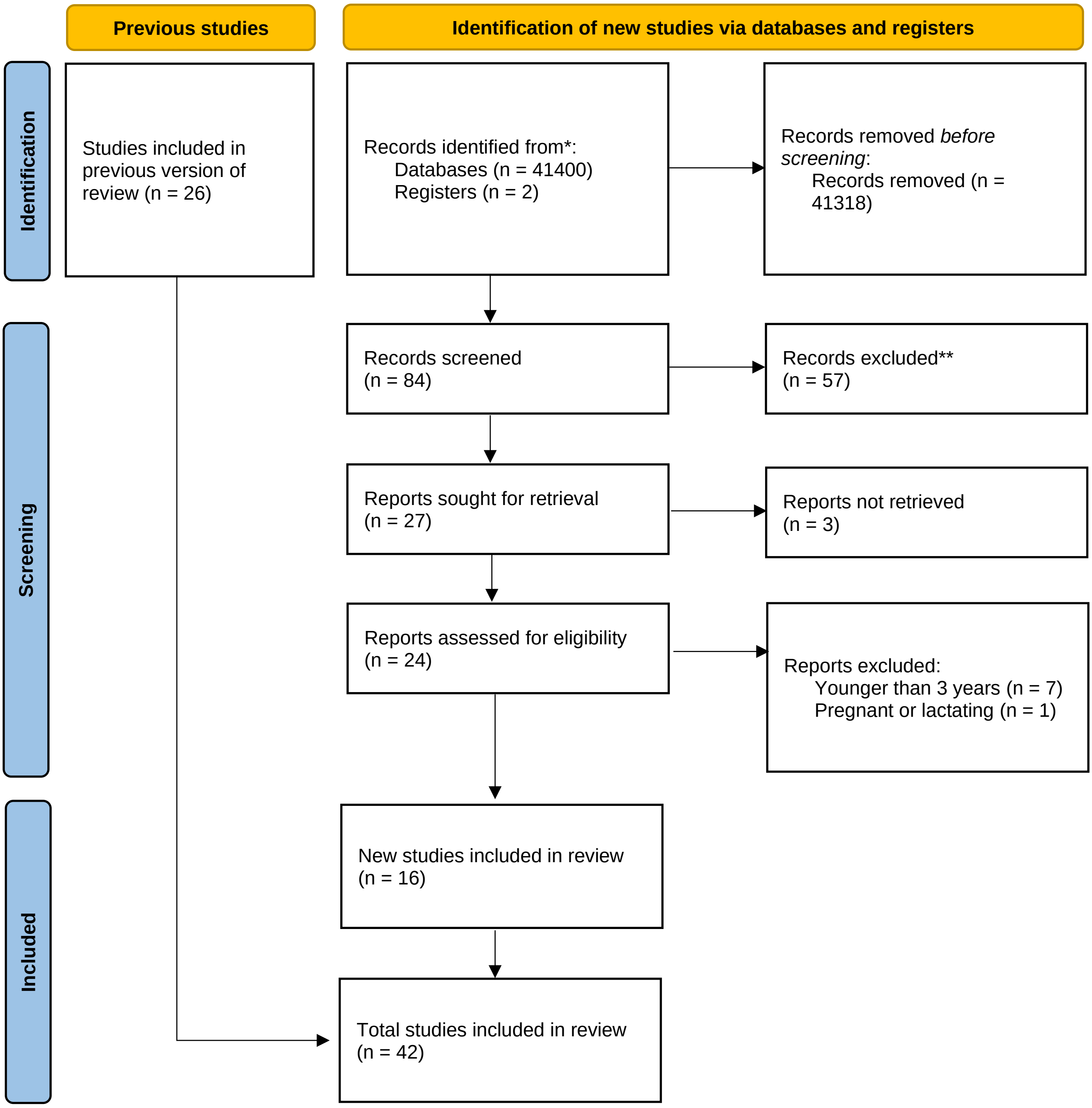

Table S1. Number of included studies and participants for each graph.

Mineral	Subject	Number of studies	Number of participants
Ca	Figure 1	37	352
	Figure 2	35	314
	Figure 3	13	75
	Figure 4	19	90
	Figure 5	17	89
	Figure 18	13	59
P	Figure 6	37	358
	Figure 7	37	358
	Figure 8	37	358
	Figure 9	12	71
	Figure 10	20	113
	Figure 11	19	85
	Figure 12	17	105
Mg	Figure 13	35	326
	Figure 14	34	318
	Figure 15	14	93
	Figure 16	26	176
	Figure 17	25	171
